# Examining the Neurobiology of Non-Suicidal Self-Injury in Children and Adolescents: The Role of Reward Responsivity

**DOI:** 10.3390/jcm10163561

**Published:** 2021-08-13

**Authors:** Julia A. C. Case, Matthew Mattoni, Thomas M. Olino

**Affiliations:** Department of Psychology, College of Liberal Arts, Temple University, 1701 North 13th Street, Weiss Hall, Philadelphia, PA 191221, USA; matt.mattoni@temple.edu (M.M.); thomas.olino@temple.edu (T.M.O.)

**Keywords:** non-suicidal self-injury, reward processing, depression, functional magnetic resonance imaging, functional connectivity

## Abstract

Although prior work has shown heightened response to negative outcomes and reduced response to positive outcomes in youth with a history of non-suicidal self-injury (NSSI), little is known about the neural processes underlying these responses. Thus, this study examined associations between NSSI engagement and functional activation in specific regions of interest (ROIs) and whole-brain connectivity between striatal, frontal, and limbic region seeds during monetary and social reward tasks. To test for specificity of the influence of NSSI, analyses were conducted with and without depressive symptoms as a covariate. We found that NSSI was associated with decreased activation following monetary gains in all ROIs, even after controlling for depressive symptoms. Exploratory connectivity analyses found that NSSI was associated with differential connectivity between regions including the DS, vmPFC, insula, and parietal operculum cortex when controlling for depressive symptoms. Disrupted connectivity between these regions could suggest altered inhibitory control of emotions and pain processing in individuals with NSSI. Findings suggest dysfunctional reward processes in youth with NSSI, even very early in the course of the behavior.

## 1. Introduction

Non-suicidal self-injury (NSSI), defined as the deliberate damage or destruction of body tissue without the intent to die, is a common behavior amongst adolescents [1,2]. Despite multiple negative sequalae of NSSI, including disruptions in interpersonal relationships and increased risk for problem behaviors including substance use, violence towards others, and suicidal attempts and deaths by suicide [3,4,5], there are only a small number of studies that address neural correlates of NSSI early in the course of these behaviors. Studies examining these correlates early in the course of NSSI may provide valuable data on processes that help distinguish between risk factors and correlates of NSSI.

Much research on NSSI has been conducted in older samples, including college-aged students or adults [3,4,5]. However, rates of NSSI are already substantial much earlier in development. NSSI is present in individuals as young as 7 [6], and epidemiological research has found that rates of NSSI increase dramatically from early adolescence (7%; [7]) to young adulthood (38%; [8]). Thus, studies of older adolescents rely on longer-term retrospective reports of the onset of behaviors, as well as thoughts, affect, and behavior surrounding the onset of NSSI. In trying to understand more about the emergence and onset of these behaviors, research involving youth nearer to the onset of NSSI may lead to better clarity between the risk and correlates of these behaviors.

### 1.1. NSSI and Associations with Negative and Positive Outcomes

Although there are many factors that are associated with NSSI engagement [9,10,11,12,13], many models propose that NSSI is used to regulate negative emotions. Indeed, a heightened experience of negative emotions following negative experiences is frequently and robustly associated with consequent NSSI [14,15,16]. Additionally, NSSI is associated with multiple dimensions of negative affect, including high arousal negative emotions (e.g., [17,18,19]), low arousal negative emotions (e.g., [20,21,22]), and self-focused negative emotions [23,24,25]. In addition to a heightened experience of negative emotions, NSSI is associated with multiple other domains associated with negative emotions, including self-reported threat sensitivity, conflict, and rejection [5,26,27].

Studies examining associations between NSSI and responses to positive outcomes report more mixed results than those between NSSI and responses to negative outcomes. Prior work has shown that individuals with NSSI rate positively-valenced images as less pleasant and, following acute stress, display reduced physiological reactivity (i.e., skin conductance) to positive images compared to individuals without NSSI [28]. Positive affect is associated with a decreased likelihood of NSSI and has been shown to moderate the relationship between negative affect, brooding, self-criticism, and NSSI, such that there is a stronger relationship between these constructs and NSSI at lower levels of positive affect [29,30]. In contrast, other work has shown that higher reward sensitivity is associated with NSSI [31,32]. Given these inconsistencies in research on responses to positive outcomes in NSSI, more research in this area is needed to elucidate the directionality of these associations.

### 1.2. Neural Correlates of NSSI

Much of the work in affective processes in NSSI has focused on self-report and behavioral responses, with the understanding of neural correlates being much less extensively examined. Preliminary work has examined NSSI in relation to negative and positive outcomes, implicating neural regions involved in reward processing, social processing, and emotional processing (see [33] for a review). For responses to negative outcomes, the few studies examining neural processes associated with NSSI in adolescent samples have shown that NSSI is associated with heightened activation in the dorsal striatum, medial prefrontal cortex, ventrolateral prefrontal cortex, and insula during social exclusion [26,34], as well as with heightened response to monetary losses versus rewards when examining feedback negativity [35]. Although no studies have been conducted in adolescents examining functional connectivity following negative outcomes, studies in adults with NSSI examining connectivity have shown negative associations between NSSI and connectivity in limbic-striatal [36] and frontal-limbic [37] networks during tasks inducing negative affect. 

For responses to positive outcomes, the few studies examining neural processes associated with NSSI in adolescent samples have shown that NSSI is associated with heightened activation in the medial prefrontal cortex and ventrolateral prefrontal cortex during social inclusion using the Cyberball task [34]. Additionally, Vega et al. [38] showed that NSSI is associated with heightened activation in the orbitofrontal cortex during monetary reward receipt in adults. Further, this study also found reduced connectivity in frontal-limbic networks following unexpected rewards in individuals engaging in NSSI, potentially indicating an impaired ability to update reward associations in NSSI. Given the consistency in findings between self-report and imaging methods for responses to negative outcomes and the discrepancy between these methods for responses to positive outcomes, further examination of these processes in NSSI is necessary.

### 1.3. NSSI and Comorbid Depressive Symptoms

Finally, NSSI is associated with multiple forms of psychopathology, such as mood, anxiety, personality, substance abuse, and externalizing disorders [39], although these behaviors are also seen in the absence of psychiatric diagnoses [40]. Importantly, NSSI is often examined through the lenses of comorbid disorders. For instance, in children and adolescents, NSSI is commonly researched within samples meeting criteria for major depressive disorder (MDD; e.g., [41]), even though less than 50% of individuals engaging in NSSI meet criteria for comorbid major depression. These designs lead to challenges in interpreting whether associations are due to NSSI or other psychiatric diagnoses. The failure to consider such comorbid diagnoses is also a challenge in studies of neural function, as there is significant overlap between the neural correlates of depression and NSSI. Major depression is associated with heightened responses to negative outcomes, such as monetary losses and social rejection [42], as well as decreased responses to positive outcomes such as monetary gains or social acceptance [43]. Towards identifying whether associations are specific to NSSI, or are better accounted for by depression, examining associations for NSSI and depressive symptoms simultaneously is key.

### 1.4. Current Study

This study examined associations between NSSI engagement and responses to negative and positive outcomes in youth with and without lifetime engagement in NSSI. This study expands on previous imaging studies of NSSI by examining neural response to negative and positive outcomes across both monetary and social stimuli. Additionally, this study assessed these constructs across both neural function and self-report measures. Indices of neural function included task-based reactivity to negative (monetary losses and social rejection) and positive (monetary gains and social acceptance) outcomes in regions of interest (ROIs), including the ventral and dorsal striatum (VS, DS), the anterior cingulate cortex (ACC), the orbitofrontal cortex (OFC), the ventrolateral and ventromedial prefrontal cortices (vlPFC, vmPFC), and the insula. We also conducted sensitivity analyses to examine the specificity of associations for NSSI by controlling for depressive symptoms. Finally, in addition to task-based reactivity, we explored functional connectivity using whole-brain psychophysiological interactions (PPI), using bilateral DS, mPFC, and insula seed ROIs, as these regions are commonly implicated in reward, social, and emotional processing. We hypothesized that NSSI would be associated with heightened neural reactivity to both negative and positive outcomes across monetary and social domains in reward- and emotion-relevant ROIs, including the ACC, the DS and VS, the OFC, and the vlPFC, the vmPFC, and the insula. We also hypothesized that NSSI would be associated with higher self-reports of responses to both negative and positive outcomes, including peer victimization, pleasure sensitivity, and social affiliation. Given the exploratory nature of our connectivity analyses, we did not have a priori hypotheses about these associations.

## 2. Materials and Methods

### 2.1. Study Participants

Participants in the current study included individuals drawn from the Temple Adolescent Depression Study. For the current manuscript, the sample contained all individuals with at least partial baseline data on the measures of interest from the parent study. Youth between 9–13 years with a primary caregiver were eligible to participate. Beyond this, there were no additional specific inclusion criteria for participation within the current study. Youth with a history of neurological disorder, head injury, pervasive developmental disorders, and/or intellectual functioning less than 70 as assessed by the Kaufman Brief Intelligence Test, second edition [44], were ineligible for study participation. Youth also were ineligible for participation if they had a history of bipolar disorder, psychosis spectrum disorders, developmental disorders or disabilities, neurological or cardiovascular diseases that affected central nervous system blood flow, or were taking any psychotropic medications at the time of recruitment or scan. In addition, youth who could not participate in imaging assessments were not eligible for inclusion (e.g., individuals with non-removable metallic implants, braces, or with conditions such as uncorrectable vision or claustrophobia that would make completing MRI assessments unsafe). 

In total, 168 participants consented and completed the MRI portion of the study; 70 participants either did not consent to participate in the MRI portion or were not eligible to complete the MRI portion of the study due to contraindications. Of the participants who completed the MRI scan, eight were excluded for incidental findings, including arachnoid cysts (N = 2), lesions (N = 3), sinusitis (N = 2), and tumors (N = 1). For the guessing task, data were collected from 150 participants; 10 participants failed to complete the task. Additionally, 29 participants were excluded due to excessive head motion (>3 mm of movement on >75% of scans) during the Guessing Task and 24 were excluded due to having <75% behavioral compliance over the full number of trials across the task. Thus, 97 participants total (30 youth with NSSI and 67 youth without NSSI) had usable data for the Guessing Task. For the Chatroom Interact Task, data were collected from 147 participants; 10 participants failed to complete the task, and an incorrect version of the task was administered to three participants. Additionally, 26 were excluded due to excessive head motion (>3 mm of movement on >75% of scans) during the Chatroom Interact Task. As participants received feedback on all trials, no participants were excluded for failing to provide behavioral responses during this task. Thus, 121 participants total (36 youth with NSSI and 85 youth without NSSI) had interpretable brain and behavioral data for the Chatroom Interact Task. Finally, self-report data were available for 171 participants total (48 youth with NSSI and 123 youth without NSSI), inclusive of the participants included in imaging analyses; thus, we used this expanded sample in our examination of self-report measures of responses to negative and positive outcomes.

### 2.2. Measures

#### 2.2.1. NSSI Status

The Kiddie Schedule for Affective Disorders and Schizophrenia-PL DSM-IV (KSADS; [45]) is a semi-structured clinical interview that assesses current and lifetime psychiatric symptoms. KSADS interviews were completed with both the primary caretakers and the youth participants. Supplemental questions during the interview assessed the lifetime presence of any NSSI behaviors. Specifically, primary caretakers and participants were asked whether youth had ever engaged in any of the following: cutting, biting, burning, carving, pinching, pulling hair, severe scratching, banging or hitting self, interfering with wound healing, rubbing skin against a rough surface, sticking self with needles, or swallowing dangerous substances. Additionally, primary caretakers and participants were asked whether youth had ever engaged in any other self-harm behaviors. Follow-up questions were asked to ensure that endorsed behaviors did not involve intent to die, which would have constituted suicidal behavior rather than NSSI.

There were no cases where a primary caretaker endorsed NSSI on the KSADS and a participant denied engaging in these behaviors. For cases where a participant endorsed NSSI and a primary caretaker denied NSSI engagement, this information was discussed with both the participant and the primary caretaker together, in order to reach a consensus. Thirty-one participants were identified as having engaged in one or more lifetime incidents of NSSI based on the KSADS.

The Inventory of Statements about Self-Injury (ISAS; [46]) is a self-report measure that was also administered to assess the lifetime presence of any NSSI. Youth participants were asked to estimate the number of times in their life they had intentionally performed each type of NSSI behavior, including those listed above as well as a write-in option. Forty-eight participants endorsed having engaged in one or more lifetime incidents of NSSI based on the ISAS. Of these, 31 were also identified by the KSADS as having engaged in one or more lifetime incidents of NSSI. Seventeen additional participants were identified on the ISAS as having engaged in one or more lifetime incidents of NSSI; no participants who endorsed NSSI on the KSADS failed to endorse NSSI on the ISAS.

#### 2.2.2. Depressive Symptoms

The Children’s Depression Inventory (CDI; [47]) is a 27-item self-report measure assessing the cognitive, affective, and behavioral symptoms of depression in children and adolescents. Participants endorse statements about their experience of depressive symptoms on a three-point Likert scale. The CDI has been used widely to assess depressive symptom severity across both clinical and community samples [48]. In the current study, internal consistency was excellent (α = 0.90). 

#### 2.2.3. Responses to Negative and Positive Outcomes

The Peer Experiences Questionnaire (PEQ; [49]) is an 18-item self-report measure assessing bullying behaviors and violence in school-age children. The PEQ Victimization Subscale (PEQ-VS) in particular contains nine items and evaluates peer victimization or the experiences of being a target of aggressive behavior by peers. Participants rate how often they have experienced a behavior: “never,” “once or twice,” “a few times,” “about once a week,” or “a few times a week.” In this study, internal consistency was excellent (α = 0.92).

The Pleasure Scale for Children (PSC; [50]) is a 39-item self-report measure assessing anhedonic responses to rewarding events and activities in school-age children. Participants are asked to indicate on a three-point Likert scale if that activity would make them feel “very happy,” “happy,” or if it “wouldn’t matter.” In this study, internal consistency was excellent (α = 0.95). 

The Early Adolescent Temperament Questionnaire (EATQ; [51]) is a 103-item self-report measure assessing temperament and self-regulation in children and adolescents. The EATQ Pleasure Sensitivity Subscale (EATQ-PSS) contains seven items assessing the threshold of pleasure sensitivity. Items are rated on a five-point Likert scale, ranging from “Almost always untrue,” to “Almost always true.” In this study, internal consistency was good (α = 0.82). 

The Behavioral Inhibition System/Behavioral Activation System Scale (BIS/BAS; [52]) is a 24-item self-report measure assessing appetitive and aversive motives. The BAS Reward Responsiveness Subscale (BAS-RRS) in particular assesses positive response to rewards. Participants rate each item on a four-point Likert scale ranging from “Very true for me” to “Very false for me.” In this study, internal consistency was adequate (α = 0.76). 

The Anticipatory and Consummatory Interpersonal Pleasure Scale (ACIPS; [53]) is a 17-item self-report scale assessing hedonic capacity for social interaction and interpersonal engagement. Participants rate how true or false the statement is for them on a four-point Likert scale, ranging from “Really false for me” to “Really true for me.” In this study, internal consistency was excellent (α = 0.93).

The EATQ Affiliation Subscale (EATQ-AS; [51) contains 8 items and assesses desire for warmth and closeness with others, such as friends and family members. Participants rate items on a five-point Likert scale, ranging from “Almost always untrue,” to “Almost always true.” In this study, internal consistency was adequate (α = 0.79).

#### 2.2.4. fMRI Tasks

**Figure 1:**
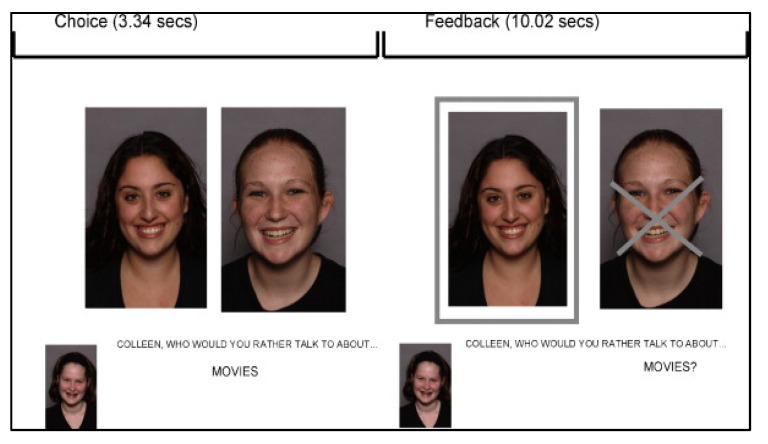
Depiction of the Chatroom Interact Task rejection trial feedback. Choice and feedback time described in seconds.

The Chatroom Interact Task examines reactions to social rejection and acceptance from virtual peers [54]. During their lab visit, participants selected preferred same-sex peers based on photographs and brief profiles. Participants also provided their own profile and photograph. During their scan visit, participants were told that they were matched with two preferred peers from the first visit and that these youth were ready to participate in an online “chat game.” During the fMRI assessment, pictures of the peers and the participant were projected on the screen two at a time, as the participant and virtual peers took turns selecting who they would rather talk to about a series of teen interests (e.g., music, friends, school). The task consisted of three experimental blocks, each with 15 trials during which a person was chosen or not chosen to discuss each topic, and a fourth motor matching control block (total run time 13 minutes, 30 s). Each block began with an instruction about who would be making choices for that block. The photograph of the agent (i.e., the ‘chooser’) was shown at the bottom left corner of the screen and the photographs of the other two players were shown next to each other in the middle of the screen. For each trial, the question, “Who would you rather talk to about…” with the selected topic for that trial (i.e., “music?”) appeared on the screen for 4 s. For the first block of the task, the participant acted as the ‘chooser.’ For blocks two and three, trials were arranged so participants experienced one block in which they were chosen on 10 of the 15 trials and another block in which they were rejected on 10 of the 15 trials. The photo of the person not chosen (i.e., “rejected”) had an X super-imposed on their image and the photo of the person who was accepted was highlighted with a border. Feedback was provided for 10 s. A fourth block was composed of trials used as motor and perceptual controls, specifically to control for viewing faces (self and others); a small gray dot appeared on one of the faces, and the participant was asked to indicate on which face the gray dot appeared. As this task was also used with participants at a later study time point, debriefing was not conducted with participants at the conclusion of the scan. Although implemented in blocks, the task was analyzed based on specific events (15 acceptance and 15 rejection trials across the task). The order of blocks and trials within blocks were randomized between participants. Participant photos were deleted from all computers at the conclusion of each assessment. See Figure 1 for a depiction of the Chatroom Interact Task.

The Guessing Task [55,56] has previously been implemented successfully in studies of young adolescents (e.g., [57,58,59]). This event-related task allows the examination of both responses to monetary gains and losses. In the task, participants guess whether the value of a card is greater than or less than five. Trial outcomes include winning $1, losing 50¢, or no change in earnings (neutral outcomes, used as control trials in the current study). The task has 48 trials and lasts for approximately 9 minutes. The sequence of trial outcomes is predetermined, allowing for a similar experience of rewarding outcomes in the same order for all participants. Participants were told that the outcomes of trials are the result of chance because striatal response to reward occurs in particular to unpredicted reward [60]. In the current study, analyses focused on responses to rewarding (and loss) outcomes.

### 2.3. Procedure

#### 2.3.1. Laboratory Visit

The Institutional Review Board at Temple University approved all study procedures, and prior to data collection, parents and children provided informed consent and assent, respectively. Youth participants completed questionnaire and interview measures at the lab visit. After collection of questionnaire and interview data, youth participants completed brief self-report forms and selected their preferences for the chatroom fMRI task. Participants also received training on tasks administered at the fMRI visit, as well as training on remaining still during the scan in a mock scanner. 

#### 2.3.2. Scan Visit

Before entering the scanner, participants completed an MRI safety screener. Tasks were presented using E-prime software [61]. Neuroimaging data were acquired using a wide bore (70 cms) 3T Philips Ingenia scanner equipped with echo planar imaging (EPI) capability suitable for functional and structural imaging with a standard 12-channel head coil located at the Jefferson Hospital Integrated Magnetic Resonance Imaging Center. Sessions began with a 10-s scout to ensure proper head position. Structural 3D axial MPRAGE images were acquired (1 mm thick; TR = 2200 ms; TE = 3.29 ms; FOV = 256 × 256; Matrix = 256 × 256; Flip Angle = 9°; 192 slices). BOLD functional images were acquired with a gradient echo planar imaging sequence and covered 34 axial slices (3 mm thick) beginning at the cerebral vertex and encompassing the entire cerebrum and the majority of the cerebellum (TR/TE = 2000/25 ms, field of view = 20 cm, matrix = 64 × 64). Scanning parameters were selected to optimize BOLD signal quality while maintaining a sufficient number of slices to acquire whole-brain data. Scanning was synchronized to stimulus presentation, and multiple images were obtained during the course of a trial. Before the collection of fMRI data for each participant, a reference echo planar imaging scan was acquired and visually inspected for artifacts (e.g., ghosting) and good signal across the entire volume. Total scan time was approximately one hour. During the scan acquisition, tasks were administrated in a fixed order, where the Chatroom Interact Task was administered prior to the Guessing Task for all participants except for three, due to administrator error.

### 2.4. Data Analysis

#### 2.4.1. Imaging Preprocessing

Data preprocessing was conducted with Statistical Parametric Mapping Version 12 (SPM 12; [62]) and was facilitated by the Functional Connectivity Toolbox [63] using standard procedures. For each scan, images for each participant were realigned to the first volume in the time series to correct for head motion. Realigned images were spatially normalized into Montreal Neurological Institute (MNI) stereotactic space using a 12-parameter affine model, then smoothed to minimize noise and residual difference in gyral anatomy with a Gaussian filter set at 6 mm full width at half-maximum. Voxel-wise signal intensities were ratio normalized to the whole-brain global mean. A first-level fixed-effect model was constructed for each participant and predetermined condition effects at each voxel were calculated using a t-statistic. Analyses for responses to negative outcomes focused on contrasts of loss > control for the Guessing Task and rejection > control for the Chatroom Interact Task. Analyses for responses to positive outcomes focused on contrasts of gain > control for the Guessing Task and acceptance > control for the Chatroom Interact Task. 

#### 2.4.2. Description of Regions of Interest (ROIs)

We examined regions of interest (ROIs) based on previous studies investigating neural functioning in NSSI. ROIs were defined bilaterally and obtained from the NeuroImaging Tools and Resources Collaboratory WFU_PickAtlas Toolbox, Version 3.0.3, based on the Talairach Daemon database. This approach has previously been utilized in other neuroimaging studies in adolescent populations (e.g., [43]). All realigned images were spatially normalized to the MNI echo-planar imaging template in SPM12. ROIs included the ACC, DS, VS, OFC, vlPFC, vmPFC, and insula. Mean activation for each ROI was extracted and used in analyses in external statistical analysis programs.

#### 2.4.3. ROI Analyses

A first-level model was constructed for each participant in which the hemodynamic signal was deconvolved using a general linear model. At the second-level analysis, first-level voxel-wise t-statistics were generated for each participant to calculate the following contrasts: loss > neutral outcome control (Guessing Task) and rejection > motor and perceptual control (Chatroom Interact Task) for responses to negative outcomes; and gain > neutral outcome control (Guessing Task) and acceptance > motor and perceptual control (Chatroom Interact Task) for responses to positive outcomes. For second-level analyses, parameter estimates (beta estimates) were extracted from the predefined ROIs (ACC, DS, VS, OFC, vlPFC, vmPFC, and insula). The averaged parameter value from across the entire ROI was then exported into external statistical analysis programs for further analyses. Specifically, ROI parameter estimates were used as dependent variables in linear models evaluating differences between youth with and without NSSI, both independently and when controlling for dimensional scores of depression. Due to the number of analyses, results were FDR-corrected using the Benjamini–Hochberg procedure [64]. Finally, to complement focal ROI analyses, we also conducted whole-brain post hoc analyses comparing youth with and without NSSI in the contrasts of interest for each imaging task.

#### 2.4.4. Exploratory Connectivity Analyses

Generalized psychophysiological interaction (PPI) analyses examined associations between NSSI and whole-brain connectivity for seed ROIs. PPI is used to examine context-specific changes in connectivity. This method identifies correlated activation between seed regions and other brain regions and was used previously in the other study of reward-related connectivity in NSSI (e.g., [38]). For first-level analyses, time courses of activity were extracted from the ROI seed mask and then deconvolved from the hemodynamic response function. The seeds were bilateral caudate and putamen (DS), mPFC, and insula ROIs, defined by the FSL Harvard–Oxford atlas. Time course was entered as a regressor into a GLM analysis, along with regressors for each task condition and interaction terms, consisting of the product of the task regressor multiplied by the time-series regressor for each condition. This method was selected in order to reveal regions that demonstrated activity in sync with the original seed ROI, rather than whether there was any activity more generally in these areas. PPI analyses were run for responses to negative outcomes using the Guessing Task loss > control contrast and the Chatroom Interact Task rejection > control contrast; and for responses to positive outcomes using the Guessing Task gain > control contrast and the Chatroom Interact Task acceptance > control contrast, both independently and when controlling for dimensional scores of depression. PPI connectivity analyses were cluster-level family-wise error corrected at *p* < 0.05, as implemented in the Functional Connectivity Toolbox.

#### 2.4.5. Self-Report Analyses

Youth with and without NSSI were also compared on self-report outcome measures, including the PSC, the BAS Reward Responsiveness Subscale, the EATQ-Pleasure Sensitivity Subscale, the ACIPS, the EATQ-Affiliation Subscale, and the PEQ Victimization Subscale. Each measure was used as a dependent variable in linear models evaluating differences between youth with and without NSSI, both independently and when controlling for dimensional scores of depression.

## 3. Results

Demographic and clinical characteristics of the sample are presented in Table 1. There were no significant differences in demographic and clinical characteristics between individuals with usable imaging data compared to those included in self-report analyses but not included in imaging analyses. There were also no differences in demographic and clinical characteristics between individuals based on whether they were identified by the KSADS and ISAS (N = 31) or identified solely by the ISAS (N = 17; Supplemental Table S1). Given that there were no significant differences between these features based on method of identification, we included participants within our NSSI group who had endorsed NSSI on either/both the KSADS and ISAS. Within our NSSI group, the average number of NSSI events was 60.08 (SD = 417.64; Range: 1–5000) and the average number of NSSI methods used was 2.20 (SD = 1.96; Range: 1–9). 

### 3.1. Results of ROI Analyses

#### 3.1.1. Responses to Negative Outcomes

**Figure 2:**
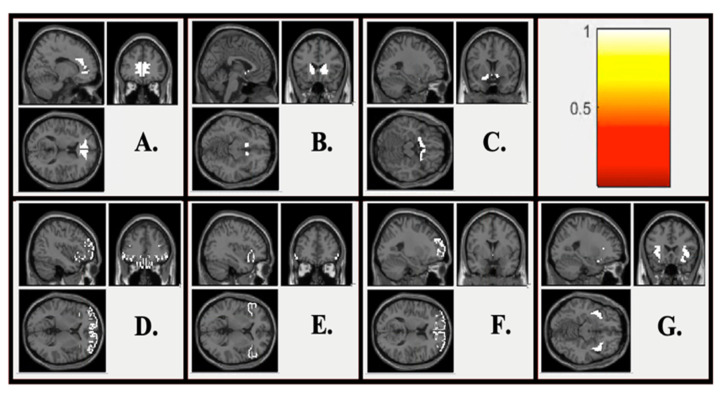
Region of Interest (ROI) maps. (**A**) Anterior cingulate cortex ROI; (**B**) Dorsal striatum ROI; (**C**) Ventral striatum ROI; (**D**) Orbitofrontal cortex ROI; (**E**) Ventrolateral prefrontal cortex ROI; (**F**) Ventromedial prefrontal cortex ROI; (**G**) Insula ROI.

Figure 2 displays ROI maps used within the current study. Linear models examined differences between youth with and without NSSI on neural responses to negative outcomes (Table 2). There were no significant differences between youth with and without NSSI on neural responses to monetary losses in any ROIs (Table 2). Additionally, there were no significant differences between youth with and without NSSI on neural responses to social rejection in any ROIs. Associations were also not significant when controlling for depressive symptoms (Supplemental Table S2).

#### 3.1.2. Responses to Positive Outcomes

Linear models examined differences between youth with and without NSSI on neural responses to positive outcomes (Table 2). There were significant differences, such that youth with NSSI displayed significantly less neural activation following monetary gains than youth without NSSI in all ROIs. Further, these results remained significant when controlling for depressive symptoms (Supplemental Table S2; Supplemental Figure S1). There were no significant differences between youth with and without NSSI on neural responses to social acceptance in any ROIs (Table 2); associations also were not significant when controlling for depressive symptoms (Supplemental Table S2). Finally, results from post hoc whole-brain analyses are described in Supplemental Section S1.

### 3.2. Results of Exploratory Connectivity Analyses

**Figure 3:**
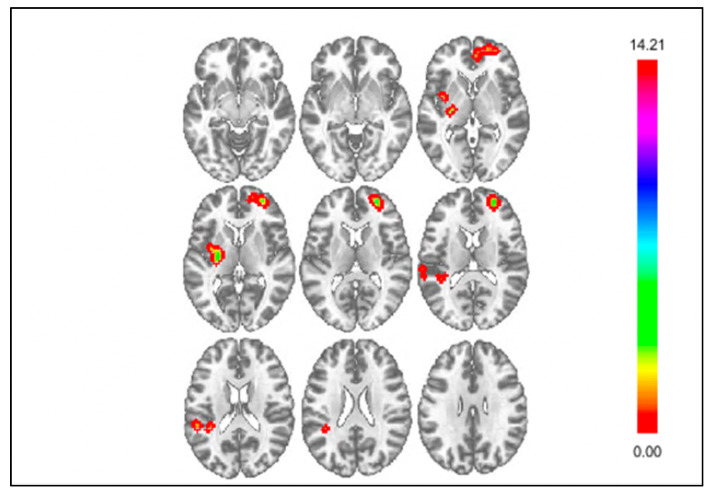
Whole brain results showing significant clusters for connectivity with the DS seed for negative outcomes, loss > control.

Exploratory whole-brain functional connectivity analyses examined differences in connectivity during responses to negative outcomes between youth with and without NSSI (Table 3). For loss > control, whole-brain analyses showed significant differences between youth with and without NSSI in connectivity between the DS and the vmPFC, insula, and parietal operculum cortex (POC). Figure 3 displays clusters with significantly different connectivity for the DS seed for loss > control. Youth with NSSI displayed attenuated negative connectivity between the DS and vmPFC compared to youth without NSSI. Youth with NSSI displayed attenuated positive connectivity between the DS and insula compared to youth without NSSI. Finally, youth with NSSI displayed negative connectivity, whereas youth without NSSI displayed positive connectivity between the DS and POC. When controlling for depressive symptoms (Supplemental Table S3), differences between youth with and without NSSI in connectivity between the DS and vmPFC, and DS and insula remained significant; however, the difference in connectivity between the DS and POC was no longer significant. Additionally, youth with NSSI displayed attenuated negative connectivity between the insula and the supramarginal gyrus compared to youth without NSSI; however, this difference was no longer significant when controlling for depressive symptoms. Finally, there were no significant differences in connectivity between youth with and without NSSI using the mPFC seed for loss > control. However, when controlling for depressive symptoms, analyses found youth with NSSI displayed stronger positive connectivity between the mPFC and the cingulate cortex than youth without NSSI. 

For rejection > control, youth with NSSI displayed attenuated positive connectivity between DS and the cerebellum compared to youth without NSSI. Additionally, youth with NSSI displayed attenuated negative connectivity between the mPFC and the central opercular cortex compared to youth without NSSI. These differences did not remain significant when controlling for depressive symptoms. Finally, there were no significant differences between youth with and without NSSI in connectivity using the insula seed for rejection > control with or without controlling for depressive symptoms.

**Figure 4:**
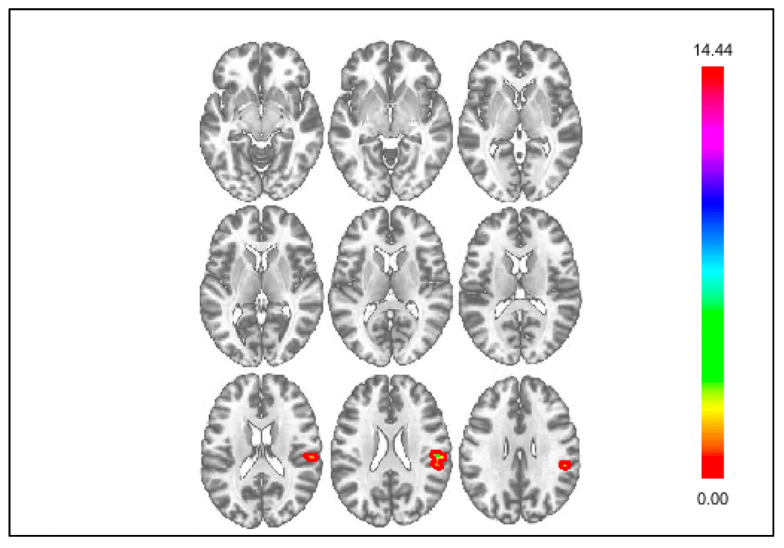
Whole brain results showing significant clusters for connectivity with the DS seed for positive outcomes, gain > control.

Whole-brain analyses also examined differences in connectivity during responses to positive outcomes between youth with and without NSSI (Table 3). For gain > control, youth with NSSI displayed negative connectivity, whereas youth without NSSI displayed positive connectivity between the DS and the POC (Figure 4). This difference remained significant when controlling for depressive symptoms (Supplemental Table S3). Additionally, youth with NSSI displayed attenuated positive connectivity between the insula and the vmPFC compared to youth without NSSI. This difference was no longer significant when controlling for depressive symptoms (Supplemental Table S3). However, when controlling for depressive symptoms, youth with NSSI displayed negative connectivity, whereas youth without NSSI displayed positive connectivity, between the insula and the temporal occipital fusiform cortex. Finally, there were no significant differences between youth with and without NSSI in connectivity between groups using the mPFC seed for gain > control with or without controlling for depressive symptoms.

Lastly, for acceptance > control, youth with NSSI displayed stronger negative connectivity between the mPFC and the POC than youth without NSSI. This association was no longer significant when controlling for depressive symptoms. However, when controlling for depressive symptoms, several other significant clusters emerged for the mPFC seed, including the planum temporale, the precuneous cortex, and the cingulate cortex (Supplemental Table S3). Specifically, youth with NSSI displayed stronger negative connectivity than youth without NSSI between the mPFC and these additional regions. Additionally, youth with NSSI displayed attenuated negative connectivity between the insula and the DS compared to youth without NSSI. This difference remained significant when controlling for depressive symptoms (Supplemental Table S3). Finally, there were no significant differences between youth with and without NSSI in connectivity using the DS seed for acceptance > control.

### 3.3. Results of Self-Report Analyses

Linear models examined differences between youth with and without NSSI on experiences of peer victimization, pleasure sensitivity, and social affiliation (Table 4). Analyses were also estimated when controlling for dimensional scores of depression (Supplemental Table S4). There were no significant differences between youth with and without NSSI on any of the self-report measures without or including depressive symptoms as a covariate.

## 4. Discussion

This study examined associations between NSSI engagement and responses to negative and positive outcomes in youth with and without lifetime engagement in NSSI. Results found that NSSI was associated with decreased activation following monetary gains in the ACC, DS, VS, OFC, vlPFC, vmPFC, and insula. These differences remained significant when controlling for symptoms of depression. Although significant differences were found following monetary gains, there were not significant group differences in activation following monetary losses or following social acceptance or social rejection. Additionally, no significant differences were found between youth with and without NSSI on self-report measures of experiences of peer victimization, pleasure sensitivity, or affiliation. Finally, exploratory analyses of connectivity found that NSSI was associated with differential connectivity between regions including the DS, vmPFC, insula, POC, supramarginal gyrus, cerebellum, and central opercular cortex. Several of these differences in connectivity remained significant when including depressive symptoms as a covariate (e.g., connectivity between the DS and vmPFC, the DS and insula, and the DS and POC). Thus, these results suggest that individuals who engage in NSSI display decreased neural responses to monetary rewards, as well as disrupted connectivity between frontal, reward, and emotion-related regions of the brain.

We hypothesized that NSSI would be associated with heightened reactivity to monetary gains in reward-relevant ROIs. Our hypotheses were based on a small number of studies of NSSI and reward processing that examined different stages of reward processing or different reward modalities. For instance, Sauder et al. [65] found that NSSI was associated with decreased activation during reward anticipation in the putamen, OFC, and amygdala. Additionally, using an event-related monetary gambling task, Vega et al. [38] found that NSSI was associated with heightened activation in the OFC during monetary reward receipt. However, this finding was only significant when examining reward prediction error trials. No differences were found in activation for expected rewards. However, our findings showed that NSSI was associated with decreased activation following monetary gains in reward-relevant ROIs. Thus, it is possible that our findings differed from these previous studies due to our examination of responses to rewards of an expected value rather than reward anticipation or receipt of unexpected rewards. 

Our findings of decreased activation following monetary gains in NSSI in reward-related regions, including the ACC, DS, VS, OFC, vlPFC, vmPFC, and insula, parallel findings of hyporesponsivity to rewards in MDD. However, our findings showed that the associations for NSSI were significant beyond the effect of depressive symptoms. Decreased activation within reward-related regions suggests that compromised reward processes, such as reward valuation or learning, could contribute to NSSI. One explanation for these results is that hyposensitivity to rewards leads individuals to seek out behaviors they believe will elicit or provoke reward responses, such as NSSI or other “risky” behaviors (e.g., alcohol or substance use), as a means of upregulating hedonic responses (e.g., [66]).

We did not find significant differences in activation between groups following monetary losses. This result was consistent with the one previous study that did not find group differences in monetary loss anticipation between individuals with and without NSSI in the OFC, striatum, or amygdala [65]. We also did not find differences in activation following social acceptance or social rejection, which was somewhat surprising given previous findings (e.g., [26,34]). Our lack of significant findings for neural responses to negative and positive social outcomes could potentially be due to the young age of our sample. Specifically, our sample may not have developed enough into adolescence to show greater social reorientation [67,68]. Perhaps assessments later in adolescence would yield stronger effects for social feedback; this is an area for future work. 

There were non-significant differences between youth with and without NSSI on self-report measures of experiences of peer victimization, pleasure sensitivity, and social affiliation. This suggests that although youth with NSSI display differential neural responses to positive outcomes, these differences are not seen when utilizing alternate methods, such as self-report scales. It is possible that the neural differences seen between youth with and without NSSI may not be affecting their behavior, as reflected by their self-reports. Alternatively, obtaining differences in self-report measures may require greater accumulation of NSSI behaviors. Thus, these associations may be stronger later in development.

Beyond regional activation, our exploratory connectivity analyses found differential connectivity between groups following negative and positive outcomes between regions including the DS, vmPFC, insula, supramarginal gyrus, POC, cerebellum, and central operculum cortex. The vmPFC and supramarginal gyrus have been implicated in regulating emotional responses to situations [69,70], the insula and supramarginal gyrus are involved in emotion processing and arousal [71], and although the cerebellum is primarily known for being responsible for coordinating voluntary movements and motor skills, such as balance, coordination, and posture [72], more recent work has discovered that the cerebellum also plays a key role in the experience and regulation of emotional states in relation to motor, cognitive, and social behaviors [73]. Thus, disrupted connectivity with these regions could suggest deficits in inhibitory control of emotions in individuals with NSSI. Additionally, the POC has previously been implicated in pain perception in humans [74,75], and the central opercular cortex covers the upper part of the insular lobe and is thought to play a role in thermosensory and nociceptive processes [76]; as such, disrupted connectivity within these regions may suggest altered pain processing in NSSI. Notably, findings of disrupted connectivity between the DS and vmPFC, DS and insula, and DS and POC remained significant when controlling for depressive symptoms, suggesting that these differences are specific to NSSI rather than due to symptoms of depression.

The current study extends the literature in a number of important ways. First, we examined associations between NSSI and responses to both negative and positive outcomes across domains of reward. This allowed for the examination of whether NSSI involves altered responses to these outcomes globally, or if it is limited to specific domains or valences of outcome. Second, previous imaging studies of NSSI have predominantly studied NSSI in the presence of comorbid psychopathology [26,34,38], but have not systematically included comorbidities or dimensional symptoms as well within analyses. We examined associations controlling for depressive symptoms and found generally consistent patterns of results with and without considering depressive symptoms.

There were, however, several limitations to this work. First, the NSSI group was heterogeneous based on both chronicity and severity of NSSI. We defined NSSI engagement as one or more lifetime NSSI events, which was inclusive of youth following both chronic and experimental courses. These heterogeneous patterns may be characterized by different neural and/or behavioral patterns that may have weakened findings. Second, although we examined NSSI nearer to the onset or emergence of NSSI than in previous studies, this study examined differential responses to negative and positive outcomes following NSSI onset. Thus, it remains unknown whether these differences in functional activation and connectivity precede or follow NSSI onset. Only one study has taken a prospective approach [77], finding that thoughts of NSSI were associated with altered reward processing in response to monetary rewards in a sample of adolescents at risk for future engagement for NSSI but who had yet to actually engage in these behaviors. Future additional work utilizing similar prospective study designs can examine those associations in youth with stronger implications for prevention efforts. Third, the current study did not counter-balance the presentation of tasks between subjects during imaging acquisition. This could have contributed to a reward-framing effect [78] that may have reduced the power to detect differences in activation in the Chatroom Interact Task, which was presented as the second behavioral task for the vast majority of participants. Future studies utilizing multiple reward tasks should counter-balance and randomize task presentation based on reward domain (monetary vs. social) to enhance the interpretational leverage of their findings.

## 5. Conclusions

NSSI behaviors are common in youth with and without psychopathology and are associated with many adverse outcomes. Although prior work has shown differential responses to negative and positive outcomes in NSSI, little is known about the neural processes involved in these findings. We found that NSSI was associated with decreased activation following monetary gains in the ACC, DS, VS, OFC, vlPFC, vmPFC, and insula. Further, these differences remained significant when controlling for depressive symptoms, suggesting that these associations are specific to NSSI. Finally, exploratory connectivity analyses found that NSSI was associated with differential connectivity between regions, including the DS, vmPFC, insula, and POC, when controlling for depressive symptoms. These activation and connectivity findings suggest altered emotional and pain processing in NSSI in early adolescence, and contribute to the literature examining reward processes in NSSI across domains in the youngest sample of individuals engaging in NSSI to date. Suggestions for future work include more stringent requirements for NSSI inclusion, as well as the examination of these features prior to the onset of NSSI, in order to contribute to the prediction and prevention efforts of these behaviors in youth. 

## Figures and Tables

**Table 1:** Demographic and clinical characteristics in youth engaging and not engaging in NSSI.

	No NSSI	NSSI
Age at Lab Visit, years M (SD)	11.01 (1.48)	11.03 (1.49)
Age at Scan Visit, years M (SD)	11.32 (1.45)	11.53 (1.54)
Time Between Lab Visit and Scan Visit, months M (SD)	4.50 (2.42)	5.10 (2.65)
Female	58.3%	54.1%
Race		
White	44.6%	50.0%
Black	39.9%	36.7%
Asian	0.6%	0.0%
Multiracial	13.7%	6.7%
Other	1.2%	6.7%
Ethnicity		
Hispanic	11.3%	10.0%
Covariate		
CDI M (SD)	32.59 (6.42) **	36.17 (7.91) **
Outcomes		
PEQ-VS M (SD)	12.54 (5.20)	13.15 (6.50)
BAS-RRS M (SD)	17.03 (2.87)	17.11 (2.31)
PSC M (SD)	14.54 (6.22)	15.09 (6.32)
EATQ-PSS M (SD)	88.21 (16.82)	88.59 (15.04)
ACIPS M (SD)	57.21 (10.84)	56.62 (8.44)
EATQ-AS M (SD)	19.20 (6.27)	19.35 (5.40)

NSSI = Non-suicidal self-injury. M = Mean, SD = Standard deviation. CDI = Children’s Depression Inventory. PEQ-VS = Peer Experiences Questionnaire, Victimization Subscale. BAS-RRS = Behavioral Activation Scale, Reward Responsiveness Subscale. PSC = Pleasure Scale for Children. EATQ-PSS = Early Adolescent Temperament Questionnaire (EATQ) Pleasure Sensitivity Subscale. ACIPS = Anticipatory and Consummatory Interpersonal Pleasure Scale. EATQ-AS = EATQ, Affiliation Subscale. ** Significant differences between groups at p_FDR-corrected_ <0.01.

**Table 2:** Associations between NSSI status and neural responses to negative and positive outcomes in a priori ROIs.

	Responses to Negative Outcomes				Responses to Positive Outcomes			
	Monetary Losses		Social Rejection		Monetary Gains		Social Acceptance	
	*B* (*SE*)	*pr*	*B* (*SE*)	*pr*	*B* (*SE*)	*pr*	*B* (*SE*)	*pr*
**ACC**	−0.09 (0.37)	−0.02	−0.11 (0.23)	−0.05	−1.15 (0.41) **	−0.28	0.10 (0.23)	0.04
**DS**	−0.21 (0.35)	−0.06	0.06 (0.21)	0.03	−1.18 (0.41) **	−0.29	0.29 (0.21)	0.13
**VS**	−0.12 (0.33)	−0.04	0.01 (0.22)	0.00	−1.51 (0.47) **	−0.31	0.26 (0.22)	0.11
**OFC**	0.14 (0.37)	0.04	−0.21 (0.22)	−0.09	−1.42 (0.43) ***	−0.32	0.05 (0.21)	0.02
**vlPFC**	−0.03 (0.35)	−0.01	−0.10 (0.19)	−0.05	−1.39 (0.41) ***	−0.33	0.16 (0.19)	0.08
**vmPFC**	0.13 (0.39)	0.03	−0.03 (0.25)	−0.01	−1.04 (0.44) **	−0.24	0.14 (0.24)	0.05
**Insula**	−0.27 (0.31)	−0.09	0.10 (0.20)	0.05	−1.26 (0.41) **	−0.30	0.33 (0.19)	0.16

*B* = Unstandardized beta. *SE* = Standard error. *pr* is a measure of effect size. ACC = Anterior cingulate cortex. DS = Dorsal striatum. VS = Ventral striatum. OFC = Orbitofrontal cortex. vlPFC = Ventrolateral prefrontal cortex. vmPFC = Ventromedial prefrontal cortex. ** Significant differences between groups at p_FDR-corrected_ < 0.01. *** Significant differences between groups at p_FDR-corrected_ < 0.001.

**Table 3:** Whole-brain connectivity results: Differences between youth with and without NSSI.

**Seed**	**Cluster Location**	**Cluster Size**	**Coordinates**			**Statistic**
		k_E_	x	y	z	Z
Monetary Loss						
DS Seed	vmPFC	112	+24	+57	+12	4.47
	Insula	61	−27	−12	+06	4.29
	Parietal Operculum Cortex	55	−39	−39	+18	3.90
mPFC Seed	*n.s.*	---	---	---	---	---
Insula Seed	Supramarginal Gyrus	41	−60	−33	+42	4.00
**Seed**	**Cluster Location**	**Cluster Size**	**Coordinates**			**Statistic**
		k_E_	x	y	z	Z
Social Rejection						
DS Seed	Cerebellum	77	+06	−84	−42	4.34
mPFC Seed	Central Opercular Cortex	102	−57	−21	+15	5.12
Insula Seed	*n.s.*	---	---	---	---	---
Monetary Gain						
DS Seed	Parietal Operculum Cortex	51	+51	−21	+24	4.5
mPFC Seed	*n.s.*	---	---	---	---	---
Insula Seed	vmPFC	52	−24	+69	−06	3.92
Social Acceptance						
DS Seed	*n.s.*	---	---	---	---	---
mPFC Seed	Parietal Operculum Cortex	283	−51	−36	+15	4.63
Insula Seed	DS	53	−03	+03	+06	4.88

DS = dorsal striatal; mPFC = medial prefrontal cortex; vmPFC = ventromedial prefrontal cortex. Voxel threshold p-uncorrected at *p* < 0.001. Cluster threshold cluster-size *p*-FWE corrected at *p* < 0.05. Directionality of differences are described within the text.

**Table 4:** Associations between NSSI status (as the independent variable) and self-report responses to negative and positive outcomes.

	*B* (*SE*)	*pr*
PEQ-VS	0.61 (1.03)	0.05
BAS-RRS	0.09 (0.46)	0.01
PSC	0.38 (2.91)	0.01
EATQ-PSS	0.54 (1.13)	0.04
ACIPS	−0.58 (1.87)	−0.03
EATQ-AS	0.14 (1.09)	0.01

*B* = unstandardized beta. *SE* = standard error. *pr* is a measure of effect size. PEQ-VS = Peer Experiences Questionnaire, Victimization Subscale. BAS-RRS = Behavioral Activation Scale, Reward Responsiveness Subscale. PSC = Pleasure Scale for Children. EATQ-PSS = Early Adolescent Temperament Questionnaire (EATQ) Pleasure Sensitivity Subscale. ACIPS = Anticipatory and Consummatory Interpersonal Pleasure Scale. EATQ-AS = EATQ, Affiliation Subscale.

## Supplementary Materials

The following will be made available online at https://www.mdpi.com/article/10.3390/jcm10163561/s1, Figure S1: Whole-brain post hoc analyses for gains > control, controlling for depressive symptoms; Table S1: NSSI frequency and methods by KSADS vs. ISAS.; Table S2: Functional ROI results for neural responses to negative and positive outcomes, controlling for depressive symptoms; Table S3: Whole-brain connectivity results controlling for depression; Table S4: Self-report responses to negative and positive outcomes, controlling for depressive symptoms; Section S1: post hoc whole-brain analyses.
