# Supplementary material for: Examining the Neurobiology of Non-Suicidal Self-Injury in Children and Adolescents: The Role of Reward Responsivity
Source: J Clin Med. 2021 Aug 13;10(16):3561. doi: 10.3390/jcm10163561 (PMC8396887; doi:10.3390/jcm10163561)

Supplemental Table S1. *NSSI frequency and methods by KSADS vs. ISAS.*

Method	Endorsed on KSADS	Endorsed on ISAS	Number Endorsed Across Measures	Number of NSSI Events M (SD)	Range of Events [min, max]
Cutting	21	21	21	3.96 (1.66)	[1, 5]
Severe Scratching	6	13	13	27.15 (35.83)	[2, 104]
Banging or Hitting	14	18	18	36.52 (108.95)	[1, 500]
Biting	2	11	11	85.85 (275.03)	[1, 1000]
Burning	1	1	1	1 (*n/a*)	---
Interfering with Wound Healing	1	18	18	43.90 (54.30)	[2, 200]
Carving	0	0	0	---	---
Rubbing Skin Against Rough Surface	0	6	6	54.00 (120.59)	[1, 300]
Pinching	5	16	16	17.45 (54.97)	[1, 250]
Sticking Self with Sharp Objects	0	3	3	1 (0)	---
Hair Pulling	2	14	14	20.07 (34.67)	[1, 100]
Swallow Dangerous Substances	0	2	2	1.33 (.58)	[1, 2]

NSSI = Non-suicidal self-injury. M = Mean, SD = Standard deviation.

Supplemental Table S2. *Functional ROI results for neural responses to negative and positive outcomes, controlling for depressive symptoms.*

		Responses to Negative Outcomes				Responses to Positive Outcomes			
		Monetary Losses		Social Rejection		Monetary Gains		Social Acceptance	
		B (SE)	*pr*	*B (SE)*	*pr*	*B (SE)*	*pr*	*B (SE)*	*pr*
ACC	NSSI	-.37 (.39)	-.11	-.13 (.25)	-.05	-1.42 (.44)**	-.35	.15 (.26)	.06
	Depression	-.01 (.03)	-.02	-.02 (.02)	-.09	.03 (.03)	.11	-.02 (.02)	-.08
DS	NSSI	-.49 (.36)	-.15	-.01 (.24)	-.00	-1.37 (.43)**	-.34	.27 (.24)	.12
	Depression	.00 (.03)	.00	-.01 (.02)	-.05	.05 (.03)	.18	-.01 (.02)	-.06
VS	NSSI	-.25 (.36)	-.08	.00 (.25)	.00	-1.81 (.51)***	-.35	.30 (.24)	.12
	Depression	.00 (.03)	.00	-.02 (.02)	-.09	.08 (.04)	.24	-.02 (.02)	-.11
OFC	NSSI	-.06 (.40)	-.02	-.26 (.24)	-.11	-1.68 (.46)***	-.39	.09 (.23)	.04
	Depression	-.02 (.03)	-.07	-.02 (.02)	-.13	.04 (.03)	.12	-.03 (.02)	-.15
vlPFC	NSSI	-.23 (.39)	-.07	-.10 (.22)	-.05	-1.64 (.45)***	-.39	.22 (.21)	.11
	Depression	-.01 (.03)	-.04	-.02 (.02)	-.12	.04 (.03)	.12	-.02 (.02)	-.15
vmPFC	NSSI	-.21 (.41)	-.06	-.11 (.27)	-.04	-1.28 (.46)**	-.30	.14 (.27)	.06
	Depression	-.01 (.03)	-.03	-.01 (.02)	-.06	.03 (.03)	.11	-.01 (.02)	-.06
Insula	NSSI	-.51 (.34)	-.17	.02 (.22)	.01	-1.45 (.45)**	-.35	.30 (.22)	.14
	Depression	-.01 (.03)	-.03	-.01 (.02)	-.03	.04 (.03)	.15	-.01 (.02)	-.04

ACC = Anterior cingulate cortex. DS = Dorsal striatum. VS = Ventral striatum. OFC = Orbitofrontal cortex. vlPFC = Ventrolateral prefrontal cortex. vmPFC = Ventromedial prefrontal cortex. **Significant differences between groups at $p_{FDR\text{-}corrected} < .01$ ***Significant differences between groups at $p_{FDR\text{-}corrected} < .001$.

Supplemental Table S3. *Whole-brain connectivity results controlling for depression.*

Seed	Cluster Location	Cluster Size	Coordinates			Statistic
		k_E	x	y	z	Z
		Monetary Loss				
DS Seed	vmPFC	52	+24	+57	+12	4.53
	Insula	48	-27	-12	+06	4.59
mPFC Seed	Cingulate Cortex	59	+15	+06	+33	4.28
Insula Seed	*n.s.*	---	---	---	---	---
		Social Rejection				
DS Seed	*n.s.*	---	---	---	---	---
mPFC Seed	*n.s.*	---	---	---	---	---
Insula Seed	*n.s.*	---	---	---	---	---
		Monetary Gain				
DS Seed	Parietal Operculum Cortex	65	+51	-21	+24	4.57
mPFC Seed	*n.s.*	---	---	---	---	---
Insula Seed	Temporal Occipital Fusiform Cortex	79	+36	-54	-15	4.10
		Social Acceptance				
DS Seed	*n.s.*	---	---	---	---	---
mPFC Seed	Planum Temporale Left	191	-57	-21	+15	4.12
	Precuneous Cortex	57	-03	-60	+18	4.16
	Cingulate Cortex	49	-06	+42	+09	4.18
Insula Seed	Caudate	55	-03	+03	+06	4.41
	Lateral Occipital Cortex	54	-30	-69	+06	4.36

DS = Dorsal striatal. mPFC = Medial prefrontal cortex. vmPFC = Ventromedial prefrontal cortex. Voxel threshold p-uncorrected at $p < .001$. Cluster threshold cluster-size p-FWE corrected at $p < .05$.

Supplemental Table S4. *Self-report responses to negative and positive outcomes, controlling for depressive symptoms.*

	NSSI		Depression	
	B (SE)	*pr*	*B (SE)*	*pr*
PEQ-VS	-.42 (.97)	-.04	.32 (.06)	.42
BAS-RRS	.41 (.47)	.07	-.09 (.03)	-.21
PSC	2.47 (2.96)	.07	-.54 (.19)	-.23
EATQ-PSS	1.14 (1.16)	.08	-.16 (.07)	-.18
ACIPS	1.48 (1.86)	.07	-.51 (.12)	-.35
EATQ-AS	.76 (1.11)	.06	-.18 (.07)	-.21

B = Unstandardized beta. *SE* = Standard error. *pr* is a measure of effect size. *PEQ-VS = Peer Experiences Questionnaire, Victimization Subscale. BAS-RRS = Behavioral Activation Scale, Reward Responsiveness Subscale. PSC = Pleasure Scale for Children. EATQ-PSS = Early Adolescent Temperament Questionnaire (EATQ) Pleasure Sensitivity Subscale. ACIPS = Anticipatory and Consummatory Interpersonal Pleasure Scale. EATQ-AS = EATQ, Affiliation Subscale.*

Supplemental Section S1.

To complement the focal ROI analyses, we also conducted post-hoc whole-brain analyses comparing youth with and without NSSI in the contrasts of interest. There were no significant differences between groups at $p_{FWE} < .05$ for any of the contrasts of interest. However, when controlling for depression, significant differences between groups at $p_{FWE} < .05$ emerged for the gains > control contrasts, whereby youth with NSSI displayed less activation than youth without NSSI, with clusters located in the DS, insula, ACC, VS, and OFC (see Supplemental Figure 1).

Supplemental Figure S1. *Whole-brain post-hoc analyses for gains > control, controlling for depressive symptoms.*

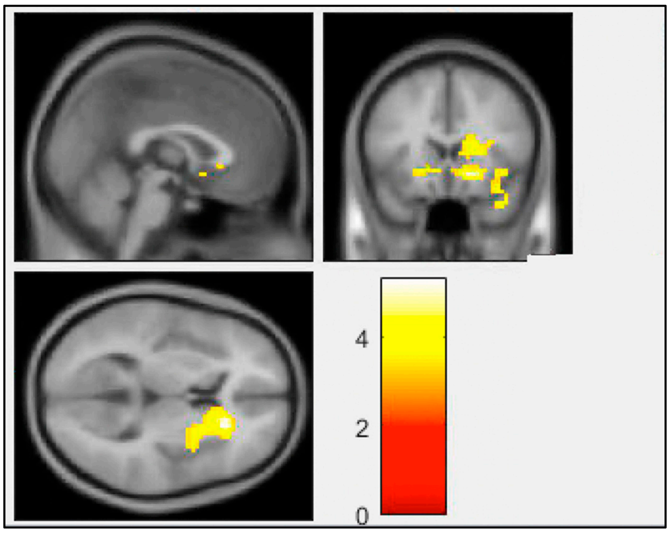

Supplement: Supplementary file 1 [file jcm-10-03561-s001.zip › jcm-1319015-supplementary.pdf]
